# Effects of dietary nutrient levels on microbial community composition and diversity in the ileal contents of pregnant Huanjiang mini-pigs

**DOI:** 10.1371/journal.pone.0172086

**Published:** 2017-02-14

**Authors:** Yujiao Ji, Xiangfeng Kong, Huawei Li, Qian Zhu, Qiuping Guo, Yulong Yin

**Affiliations:** 1 Hunan Provincial Engineering Research Center of Healthy Livestock, Key Laboratory of Agro-ecological Processes in Subtropical Region, Institute of Subtropical Agriculture, Chinese Academy of Sciences, Changsha, Hunan, China; 2 Research Center of Mini-pig, Huanjiang Observation and Research Station for Karst Ecosysterms, Huanjiang, Guangxi, China; Western University of Health Sciences, UNITED STATES

## Abstract

The mammalian gut microbiota influences various metabolic and physiological processes. Substantial metabolic changes occur during a healthy pregnancy that may be related to microbiota composition dynamics. However, the effect of diet on intestinal microbiota composition and diversity during pregnancy remains unclear. We examined the ileal contents of Huanjiang mini-pigs at two pregnancy stages to determine the effects of dietary nutrient levels on such microbial communities. Animals received either a higher-nutrient (HN) diet formulated to meet US National Research Council requirements or a lower-nutrient (LN) diet that met the Chinese National Feeding Standard recommendations. On day 45 or 75 of pregnancy, sows were euthanized and their ileal contents sampled. Experimental diet and pregnancy stage did not affect ileal bacterial richness or diversity, as determined by Chao1 and ACE species richness measures and Shannon and Simpson indices, respectively. The phyla Firmicutes and Proteobacteria, accounting for 69.99–85.44% and 5.82–15.17% of the total reads, respectively, predominated regardless of diet. At the genus level, diet significantly affected the abundance of *Lactobacillus* species, which was greater in pigs given HN feed (*P <* 0.05), but had little impact on that of *Megasphaera* species (*P* = 0.096). Pregnancy stage had a minimal effect on Proteobacteria numbers (*P* = 0.053). The number of bacteria of the phylum Firmicutes and genus *Lactobacillus* decreased, while that of the phylum Proteobacteria, family Enterobacteriaceae, and genus *Bacteroides* increased between days 45 and 75 of pregnancy. Of the short-chain fatty acids (SCFAs) measured, only propionate levels changed significantly, with higher concentrations observed on day 45 than on day 75. Our findings indicate that Firmicutes and Proteobacteria dominate pregnant sow ileal bacterial profiles. Excepting a tendency for the number of Proteobacteria to increase as pregnancy progressed, pregnancy stage and diet had little effect on ileal microbiotic composition and diversity and luminal SCFA concentrations.

## Introduction

Bacterial communities play a very important role in the biological transformation of organic matter from dietary and endogenous origins [1], and influence host metabolism and physiology [2,3]. The composition of the intestinal microbiota is affected by various factors, including the intestinal environment, nutritional and non-nutritional dietary components, and antibiotic use, among other factors [4]. For example, in mammals, proteins and their amino acid-derived metabolites can affect the relationship between the gut microbiota and intestinal mucosal morphology, metabolism, and physiology, depending on diet quality and quantity [5,6]. Surprisingly, little is known regarding the effects of pregnancy on microbial composition, diversity, and metabolic activity in the small and large intestines. In swine, these organs’ microbiotas are thought to influence health and performance. Therefore, microbial populations in various segments of the intestinal tract should be monitored. A previous study reported that colonic bacterial richness decreased in pregnant Huanjiang mini-pigs as gestational age increased. In addition, elevated nutrient levels heightened the production of metabolites related to nitrogen metabolism (Kong *et al*., unpublished data). The ileum contains a larger and more complex microbiota than the proximal sections of the small intestine (i.e., the duodenum and jejunum) [7]. Therefore, it is also important to determine changes in microbial community composition and diversity in the ileal contents of pregnant sows fed various diets.

Reproduction is clearly critical to animal farming, being the principal process for producing offspring and preserving genetic resources [8]. During a healthy pregnancy, substantial hormonal, immunological, and metabolic changes occur in the body [9]. The mammalian gut is inhabited by a complex micro-ecosystem. Moreover, recent work suggests that changes in its microbiota can cause metabolic diseases involving inflammation and obesity, and reduce insulin sensitivity [10], and pig models can be used to gain insight into such human diseases [11–13]. Gut bacterial load has been reported to increase during gestation, and microbial diversity may be modified during pregnancy [14]. Although it has been demonstrated that the gut microbiota can induce symptoms of metabolic syndrome in non-pregnant hosts, the consequences of modified host-microbiota interactions in pregnancy remain only partially characterized [15].

During pregnancy, it is common practice to maintain sows at restricted feeding levels, as excessive feeding in early pregnancy can lead to an increase in embryonic mortality [16]. In addition, increased energy intake during gestation increases the body fat content of sows, which may lead to a subsequent reduction in feed consumption during the lactation period, and cause various reproductive problems [17]. Our previous study showed that a higher-nutrient diet improves nutrient metabolism, promotes the growth and development of sows and their fetuses, and is not deleterious for reproductive performance and body composition (including fat ratio and muscle ratio) of pregnant Huanjiang mini-pigs [18]. In addition, Koren *et al*. [15] demonstrated that pregnancy was associated with profound alterations to the gut microbiota. Therefore, we hypothesized that the composition and richness of the ileal microbiota and its metabolic activity in pregnant sows might change according to dietary conditions and pregnancy stage. The present investigation was conducted to compare bacterial community composition and diversity in samples of ileal contents from Huanjiang mini-pigs fed diets with higher or lower nutrient levels from the mid- to early-late stages of pregnancy. We also analyzed levels of short-chain fatty acids (SCFAs) and branched-chain fatty acids (BCFAs), i.e., metabolites typically produced by intestinal bacteria, in the ilea of pregnant sows.

## Materials and methods

### Animals, diets, and treatments

This present study was carried out in accordance with the Chinese guidelines for animal welfare and experimental protocols, and was approved by the Animal Care and Use Committee of the Institute of Subtropical Agriculture, Chinese Academy of Sciences.

Thirty-two primiparous Huanjiang mini-pigs with a mean body weight (BW) of 46.38 ± 6.08 kg were obtained from a mini-pig farm located in Jixiang town, Huanjiang county, Guangxi province, China (108°27'40.8" E, 25°9'50" N). These sows were randomly assigned to one of the two dietary groups post-service (16 sows per dietary group and two sows per pen). One group of sows was fed a diet with a higher nutrient level (HN), while the other received feed of a lower nutrient level (LN). The HN diet was formulated to meet the nutrient recommendations of the US National Research Council [19], and contained 14.73 MJ/kg digestible energy, 13.11% crude protein, and 4.56% crude fiber. This diet is widely used in commercial crossbreed pig farms. The LN diet was formulated according to the recommendations of the Chinese National Feeding Standard for Swine, and contained 12.24 MJ/kg digestible energy, 9.77% crude protein, and 6.86% crude fiber. The LN diet is commonly used in commercial Huanjiang mini-pig farms (Table 1).

**Table 1:** Ingredients and nutritional composition of the two experimental diets (air-dried basis, %).

Ingredients^1^	HN diet	LN diet	Nutrient level^3^	HN diet	LN diet
Corn	58.20	57.20	Digestible energy (MJ/kg)	14.50	12.20
Soybean meal	11.00	0.00	Crude protein	13.10	11.00
Wheat bran	11.50	11.00	Crude protein/digestible energy	0.90	0.90
Rice bran	4.00	13.00	Crude fiber	4.56	6.86
Alfalfa meal	3.00	14.00	Ether extract	9.34	5.00
Soybean oil	7.50	0.00	Ca	0.62	0.58
Dicalcium phosphate	1.15	1.15	Total P	0.52	0.44
Limestone	0.79	0.79	Available P	0.28	0.26
Salt	0.30	0.30	Lys	1.11	0.83
Premix^2^	1.00	1.00	Met + Cys	0.65	0.52
Lys	0.88	0.88			
Met	0.27	0.27			
Thr	0.33	0.33			
Tyr	0.08	0.08			

HN, higher-nutrient; LN, lower-nutrient.

^1^ All dietary components except for the alfalfa meal were provided by a feed manufacturer (Guilin city, China), and all components were mashed and pelletized. Alfalfa meal was purchased from Gansu Tianmu Co., Ltd. (Lanzhou city, China).

^2^ Premix provided the following per kg of feed: vitamin A, 12,040 IU; vitamin D_3_, 2,112 IU; vitamin E, 29.7 IU; vitamin K_3_, 2.8 mg; vitamin B_1_, 1.2 mg; vitamin B_2_, 7.1 mg; vitamin B_6_, 1.3 mg; vitamin B_12_, 0.03 mg; nicotinic acid, 42.9 mg; pantothenic acid, 21.6 mg; folic acid, 0.44 mg; biotin, 0.12 mg; choline, 320 mg; Fe, 80 mg; Cu, 40 mg; Zn, 140 mg; Mn, 52 mg; I, 0.56 mg; Co, 1.4 mg; and Se, 0.33 mg.

^3^ Digestible energy, crude protein, Ca, total phosphorus, and available phosphorus are calculated values, while other factors are shown as measured values.

All animals were housed in 2 × 3 m pens with cement-sclerified flooring. Each pen was equipped with a feeder and a nippled drink dispenser. The room temperature was maintained at 22–28°C. All pigs had *ad libitum* access to drinking water and were fed twice daily (at 08:30 and 16:30, with approximately 2.5% of their BW) after service. All sows were checked twice daily throughout the experimental period to monitor food intake, amount of excreta, and any evidence of pain, distress, or unusual behavior.

### Sample collection

Five and eight pregnant gilts in the HN and LN diet groups, respectively, were examined 45 days after service, and six pregnant gilts in both groups were tested 75 days after service. Initially, eight sows were included in each group, but owing to unsuccessful matings, the final group numbers differed. This was addressed in our statistical analysis.

In a report by Johnston and Trottier (1999), the early, middle, and late stages of pregnancy in pigs are defined as days 1 to 30, 30 to 75, and 75 to delivery, respectively [20]. Considering the size of Huanjiang mini-pigs and the difficulty of collecting conceptus samples to determine fetal development, we chose 45 and 75 days post-service to represent the middle and early-late stages of pregnancy, respectively.

Sows were euthanized for sample collection 12 h after the last feeding on day 45 or 75 post-service [12]. Briefly, general anesthesia was induced by intravenous injection of 4% sodium pentobarbital solution (40 mg/kg BW) and euthanasia carried out by exsanguination following severing of the carotid artery [21]. The ileum was then recovered and its luminal contents collected from a region 10 cm anterior to the ileocecal valve. These were stored at −80°C for subsequent analysis of gut microbial composition and SCFA concentrations.

### Microbial DNA isolation and PCR amplification

Total bacterial DNA was extracted from ileal contents using a QIAamp DNA Stool Mini Kit (Qiagen, Hilden, Germany) according to the manufacturer's instructions. The DNA concentration of each extract was measured with a NanoDrop ND-1000 instrument (NanoDrop Technologies Inc., Wilmington, DE, USA). The 260/280 nm absorption ratio of all samples was between 1.8 and 2.0.

Bacterial community diversity and composition in each ileal sample was determined by high-throughput sequencing of microbial 16S rDNA genes. Using a previously published protocol [22], DNA was amplified by PCR with primers 515F (5′-GTGCCAGCMGCCGCGGTAA-3′) and 806R (5′-GGACTACHVGGGTWTCTAAT-3′), which target the V4 region of the 16S rRNA gene. The reverse primer contained a 6-bp error-correcting barcode unique to each sample. DNA samples were sent to a commercial service provider (Novogene, Beijing, China) for pyrosequencing on an Illumina MiSeq platform according to the manufacturer’s instructions. Raw data were obtained, before being screened and assembled using the QIIME [23] and FLASH [24] software packages. Sequencing reads were assigned to samples based on the barcodes. Reads flagged as chimeric were removed to form an “effective sequences” collection for each sample. The QIIME software package and UPARSE pipeline were used to analyze these effective sequences and determine operational taxonomic units (OTUs) [25]. Subsequently, the UCLUST algorithm [23] was employed to cluster sequences into OTUs with an identity threshold of 97%. Each OTU was assigned to a taxonomic level with RDP Classifier [26]. The sequences obtained in the present study were deposited in the National Center for Biotechnology Information Sequence Read Archive under accession numbers SRR4156412 to SRR4156415.

### SCFA and BCFA analyses

Straight-chain fatty acids, namely acetate, propionate, butyrate, and valerate, and BCFAs, namely isobutyrate and isovalerate, were analyzed as described previously [27]. To ensure their homogeneity, intestinal samples were freeze-dried using a vacuum freeze-dryer (ALPHA 2-4/LSC; Martin Christ, Osterode am Harz, Germany) at −80°C. Our preliminary data indicate that freeze-drying has little effect on the concentration of organic acids in biological samples (S1 Table). Briefly, the freeze-dried samples (0.5–0.6 g) were placed in 10-mL centrifuge tubes, mixed with 8 mL double-distilled H_2_O, homogenized, and centrifuged in sealed tubes at 7,000 × *g* at 4°C for 10 min. The resulting supernatant (0.9 mL) was mixed with 0.1 mL 25% metaphosphoric acid solution in a sealed 2-mL tube, and left to stand at 4°C for over 2 h, before being centrifuged at 20,000 × *g* at 4°C for 10 min. The supernatant was then passed through a 0.45-μm polysulfone filter and analyzed on an Agilent 6890 gas chromatograph (Agilent Technologies, Inc., Palo Alto, CA, USA) connected to a flame ionization detector and a 1.82 m × 0.2 mm (length × internal diameter) glass column packed with 10% SP-1200/1% H_3_PO_4_ on 80/100 Chromosorb W/AW (HP, Inc., Boise, ID, USA).

### Statistical analyses

Clustering and determination of alpha and beta diversity were performed in QIIME [23]. Apparent relative abundance at the phylum and genus levels, alpha diversity indices of bacterial communities, and ileal luminal SCFA concentrations were analyzed using a completely randomized design with a general linear model implemented in SAS (SAS Institute, Inc., Cary, NC, USA). Principal coordinate analysis (PCoA) of overall microbial community diversity based on an unweighted UniFrac metric was performed by the Bray-Curtis distance method to compare all samples. Rarefaction curves were created using Excel 2010 (Microsoft, Redmond, WA, USA). Phyla and genera with relative abundances below 0.5% in sows of both diet groups were excluded from further analysis. Differences were deemed statistically significant when associated with a *P*-value < 0.05. *P* < 0.10 was considered to indicate a trend toward significance.

## Results

### DNA sequence coverage and alpha diversity of bacteria from ileal contents

To assess the impact of diet and pregnancy stage on bacterial communities, sequences of the 16S rRNA gene V4 region were amplified. A total of 1,050,719 sequences (42,028.76 ± 2,520.12 per sample) were obtained, including 43,195, 42,229, 41,653, and 41,356 raw reads acquired from samples in the HN diet group on days 45 and 75 of pregnancy and those in the LN diet group at the same time points, respectively. After trimming, assembly, and quality filtering, 41,167, 40,116, 39,601, and 39,007 sequences from samples in these groups, respectively, were selected for further analysis. Considering all samples, sequence read number ranged from 32,169 to 43,472 per sample, with an average of 39,895. The average sequence read length after primer removal was 253 bp. A total of 31,460 effective sequences were extracted from each sample for comparisons at the same sequencing depth. Overall, 3,381 OTUs were detected according to a nucleotide sequence identity of 97% between reads (S2 Table).

**Fig 1:**
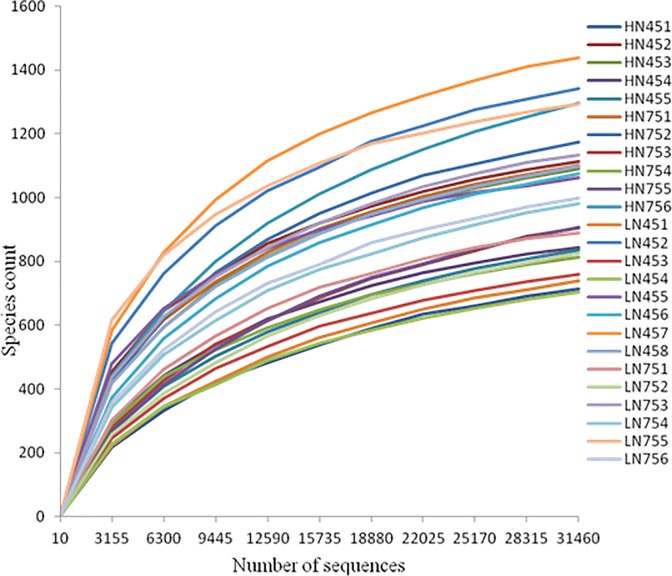
Rarefaction curves of bacterial species abundance in the ileal luminal contents of pregnant Huanjiang mini-pigs. HN45 and HN75 represent samples obtained from Huanjiang mini-pigs fed a higher-nutrient diet for 45 and 75 days, respectively. LN45 and LN75 represent samples obtained from those fed a lower-nutrient diet for 45 and 75 days, respectively.

Based on normalized subsamples of 31,460 reads per sample, rarefaction curves showed that the selected sequences were sufficient to determine the majority of bacterial diversity parameters (Fig 1). Indices of community richness (Chao1 and ACE) and diversity (Shannon and Simpson indices), with cut-off values of 0.03, are shown in Table 2. None of these measures were significantly affected by diet or pregnancy stage, although a trend toward fewer OTUs in the later stage of pregnancy (*P* = 0.083) was observed (Table 2).

**Table 2:** Alpha diversity indices of ileal bacterial communities in Huanjiang mini-pigs at different stages of pregnancy^a^.

Items	HN45	HN75	LN45	LN75	SEM	*P* values		
						Diet	Stage	Diet × Stage
OTU	1,107.80	1,247.00	1,223.38	1,208.00	44.84	0.690	0.521	0.424
Chao1	1,073.19	1,232.02	1,198.90	1,159.29	44.22	0.778	0.527	0.297
ACE	1,110.08	1,279.08	1,224.47	1,265.04	42.8	0.578	0.251	0.478
Shannon	4.81	4.94	5.31	5.47	0.01	0.126	0.284	0.802
Simpson	0.85	0.87	0.88	0.91	0.21	0.256	0.739	0.971
Coverage	99.25	99.08	99.19	99.17	0.03	0.716	0.083	0.163

^a^ Based on 31,460 reads. Here and in the following tables, HN45 and HN75 represent samples obtained from Huanjiang mini-pigs fed a higher-nutrient diet for 45 and 75 days, respectively, and LN45 and LN75 represent samples obtained from those fed a lower-nutrient diet for 45 and 75 days, respectively.

### Bacterial community composition in ileal contents

In total, genetic material from 37 bacterial phyla was identified across all ileal samples. There were six phyla with a relative abundance greater than 0.5% in at least one experimental group: Actinobacteria, Bacteroidetes, Firmicutes, Proteobacteria, Spirochaetes, and Tenericutes. Of these six phyla, Firmicutes predominated in all samples, with a relative abundance of 69.99–82.22%, followed by Proteobacteria, at 5.82–15.17%. From day 45 to 75 of pregnancy, Firmicutes abundance exhibited a decreasing trend (*P* = 0.069), whereas levels of Proteobacteria tended to increase (*P* = 0.053). Diet marginally affected the presence of Tenericutes (*P* = 0.079). However, as for the effect of pregnancy stage on Firmicutes and Proteobacteria, this change was not statistically significant (Table 3).

**Table 3:** Composition of ileal bacterial communities at the phylum level in Huanjiang mini-pigs at different pregnancy stages (%).

Items	HN45	HN75	LN45	LN75	SEM	*P* values		
						Diet	Stage	Diet × Stage
Actinobacteria	5.58	2.56	2.91	5.45	1.20	0.966	0.926	0.286
Bacteroidetes	3.08	3.05	4.97	8.06	1.28	0.205	0.568	0.562
Firmicutes	82.22	77.16	81.98	69.99	2.28	0.414	0.069	0.607
Proteobacteria	7.20	15.17	5.82	13.04	1.89	0.641	0.053	0.922
Spirochaetes	0.77	0.65	2.08	0.89	0.35	0.293	0.374	0.466
Tenericutes	0.49	0.55	0.82	1.48	0.18	0.079	0.306	0.388
Unclassified bacteria	0.15	0.18	0.26	0.24	0.03	0.242	0.920	0.711
Other bacteria	0.51	0.68	1.17	0.85	0.23	0.396	0.875	0.611

Of the 17 genera with a relative abundance greater than 0.5% in at least one of the experimental groups, only *Lactobacillus* was significantly affected by diet (*P <* 0.05). Sows fed the HN diet displayed higher numbers of sequences assigned to this genus than those given LN feed. A similar, though not statistically significant (*P* = 0.096), effect was observed in relation to *Megasphaera*. Both of these genera belong to the phylum Firmicutes. No statistically significant effect of pregnancy stage on the relative abundance of these bacteria was evident. The effect of the interaction between diet and pregnancy stage trended towards statistical significance for *Pseudomonas* (*P* = 0.080) and *Sutterella* (*P* = 0.084), members of the phylum Proteobacteria (Table 4).

**Table 4:** Composition of ileal bacterial communities at the genus level in Huanjiang mini-pigs at different stages of pregnancy (%).

Phylum	Genus	HN45	HN75	LN45	LN75	SEM	*P* values		
							Diet	Stage	Diet × Stage
Actinobacteria	*Bifidobacterium*	5.37	2.03	2.55	4.94	1.17	0.985	0.849	0.257
Bacteroidetes	*Prevotella*	0.65	0.53	0.78	1.49	0.24	0.292	0.567	0.413
Firmicutes	*Acidaminococcus*	0.86	0.03	0.02	0.01	0.16	0.194	0.204	0.211
	*Allobaculum*	0.73	0.37	0.09	0.03	0.14	0.106	0.488	0.621
	*Clostridium*	11.00	13.40	21.75	18.27	2.74	0.181	0.925	0.607
	*Dialister*	0.54	0.04	0.02	0.02	0.09	0.127	0.167	0.169
	*Lactobacillus*	29.83	20.18	12.96	4.82	3.15	0.010	0.131	0.895
	*Megasphaera*	0.55	0.21	0.10	0.10	0.08	0.096	0.320	0.295
	*Oscillospira*	0.90	0.82	1.28	1.30	0.18	0.278	0.940	0.904
	*Roseburia*	0.46	0.40	0.68	0.30	0.11	0.788	0.357	0.507
	*Ruminococcus*	1.62	1.54	2.19	2.35	0.33	0.325	0.960	0.864
	*Turicibacter*	12.47	15.35	16.89	19.16	2.47	0.442	0.630	0.955
Proteobacteria	*Acinetobacter*	0.18	0.45	0.20	2.14	0.39	0.281	0.170	0.290
	*Escherichia*	0.24	0.82	0.13	0.40	0.13	0.335	0.123	0.574
	*Pseudomonas*	0.24	0.36	0.53	0.21	0.06	0.567	0.426	0.080
	*Sutterella*	1.48	0.20	0.76	1.40	0.26	0.659	0.553	0.084
Spirochaetes	*Treponema*	0.76	0.64	2.06	0.88	0.35	0.297	0.375	0.471
Unclassified bacteria		27.90	37.54	31.94	36.90	2.64	0.759	0.197	0.673
Other bacteria		4.23	5.10	5.07	5.28	0.49	0.630	0.612	0.757

### OTU diversity

**Fig 2:**
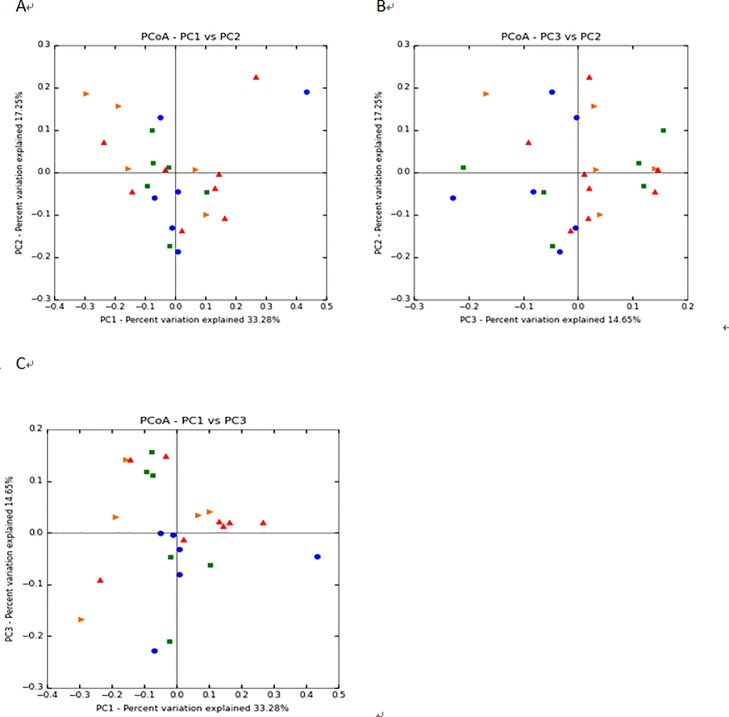
PCoA based on the UniFrac distance metric. To evaluate similarities between bacterial communities, graphs A, B, and C were generated using OTUs, based on the UniFrac distance metrics PC1 and PC2, PC3 and PC2, and PC1 and PC3 (on a two-dimensional array), respectively. Samples from each treatment group are represented as follows: ►, HN45; ■, HN75; ▲, LN45; and ●, LN75. HN45 and HN75 represent samples obtained from Huanjiang mini-pigs fed the higher-nutrient diet for 45 and 75 days, respectively. LN45 and LN75 indicate samples obtained from those given the lower-nutrient diet for 45 and 75 days, respectively.

Fig 2 shows that the bacterial communities of sows in each diet group did not substantially differ. However, those sampled on day 75 of pregnancy demonstrated a greater degree of scatter compared with those obtained on day 45.

OTUs present at <0.5% in any samples were excluded from the analysis. Of those in the phylum Firmicutes, levels of OTU-2 and OTU-906 (genus: *Lactobacillus*), OTU-45 (species: *Lactobacillus delbrueckii*), and OTU-460 (genus *Mitsuokella*) were significantly altered by diet (*P <* 0.05). All of these organisms as well as OTU-29 (species *Megasphaera elsdenii*, *P =* 0.077) were more abundant in sows fed the HN diet than in those fed the LN diet. In contrast, the presence of OTU-4 (genus *Clostridium*, *P* = 0.078), OTU-38 (order Clostridiales, *P =* 0.053), and OTU-2219 (family Clostridiaceae, *P =* 0.091) in sows given the HN diet was lower than in those fed the LN diet. Concerning pregnancy stage, levels of OTU-45 (species *Lactobacillus delbrueckii*, *P <* 0.05), OTU-906 (genus *Lactobacillus*, *P <* 0.05), and OTU-28 (family Streptococcaceae, *P =* 0.051) tended to be higher on day 45 than on day 75. In addition, OTU-10 (family Enterobacteriaceae, *P =* 0.080) and OTU-184 (genus *Bacteroides*, *P =* 0.067) were less abundant on day 45 than on day 75 of pregnancy (Table 5). A significant effect of the interaction between diet and pregnancy stage was noted for OTU-45 (species *Lactobacillus delbrueckii*, *P* < 0.05) and OTU-906 (genus *Lactobacillus*, *P* < 0.05). This effect was also evident to a certain degree, close to reaching statistical significance, for OTU-460 (genus *Mitsuokella*, *P =* 0.089).

**Table 5:** Effect of diet and pregnancy stage on OTU levels (%) in the ileal bacterial communities of Huanjiang mini-pigs*.

Items	HN45	HN75	LN45	LN75	SEM	*P* values			Annotation	Phylum
						Diet	Stage	Diet × Stage		
OTU-2	26.65	16.23	8.33	3.63	0.03	0.005	0.140	0.568	g: *Turicibacter*	Firmicutes
OTU-4	6.36	5.37	11.94	10.63	0.02	0.078	0.697	0.957	g: *Clostridium*	Firmicutes
OTU-10	3.43	10.67	2.32	7.57	0.02	0.541	0.080	0.773	f: Enterobacteriaceae	Proteobacteria
OTU-28	0.29	0.05	0.27	0.06	<0.01	0.972	0.051	0.903	f: Streptococcaceae	Firmicutes
OTU-29	0.54	0.21	0.09	0.07	<0.01	0.077	0.271	0.343	s: *Megasphaera elsdenii*	Firmicutes
OTU-38	0.12	0.09	0.21	0.26	<0.01	0.053	0.833	0.580	o: Clostridiales	Firmicutes
OTU-45	0.65	0.17	0.08	0.05	<0.01	<0.01	<0.01	<0.01	s: *Lactobacillus delbrueckii*	Firmicutes
OTU-184	0.04	0.23	0.09	0.24	<0.01	0.744	0.067	0.869	g: *Bacteroides*	Bacteroidetes
OTU-460	0.26	0.05	0.02	0.03	<0.01	0.038	0.119	0.089	g: *Mitsuokella*	Firmicutes
OTU-906	1.10	0.03	0.06	0.03	<0.01	0.013	0.010	0.014	g: *Lactobacillus*	Firmicutes
OTU-2219	0.78	0.99	1.45	1.56	<0.01	0.091	0.649	0.897	f: Clostridiaceae	Firmicutes

*Only OTUs significantly affected by diet or pregnancy stage are shown. OTUs were present at ≥ 0.5% in all cases.

f: family; g: genus; o: order; s: species.

**Fig 3:**
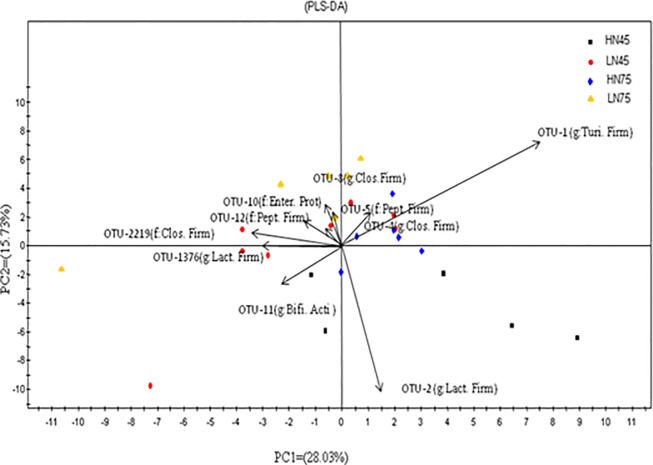
Principal component analysis of bacterial OTUs in ileal contents and the 10 predominant OTUs.

Principal component analysis at the genus level (Fig 3) revealed a tendency for each experimental group to form a distinct cluster, although some overlap was apparent. Ten dominant OTUs contributing to the variation between these groups were determined. OTU-1 and OTU-4, identified as *Turicibacter* spp. and *Clostridium* spp., respectively, were partly responsible for separating the HN diet group on day 75 of pregnancy from the other treatment groups. OTU-5 (family Peptostreptococcaceae), OTU-8 (genus *Clostridium*), OTU-10 (family Enterobacteriaceae), OTU-12 (family Peptostreptococcaceae), and OTU-2219 (family Clostridiaceae) partially distinguished the LN diet group on day 75 of pregnancy and the HN diet group on day 45 of pregnancy from the three other experimental groups. OTU-1376, OTU-11, and OTU-2 were identified as *Lactobacillus* spp., *Bifidobacterium* spp., and *Lactobacillus* spp., respectively. In addition, samples from the LN diet group on day 45 of pregnancy were more widely dispersed compared with those of other groups.

Partial least squares-discriminant analysis of bacterial OTUs in ileal contents, using diet and pregnancy stage as factors. The 10 OTUs principally responsible for the separation of samples are shown as follows: OTU-1, genus: *Turicibacter*, phylum: Firmicutes; OTU-2, genus: *Lactobacillus*, phylum: Firmicutes; OTU-4, genus: *Clostridium*, phylum: Firmicutes; OTU-5, family: Peptostreptococcaceae, phylum: Firmicutes; OTU-8, genus: *Clostridium*, phylum: Firmicutes; OTU-10, family Enterobacteriaceae, phylum: Proteobacteria; OTU-11, genus: *Bifidobacterium*, phylum: Actinobacteria; OTU-12, family: Peptostreptococcaceae, phylum: Firmicutes; OTU-1376, genus *Lactobacillus*, phylum: Firmicutes; OTU-2219, family: Clostridiaceae, phylum: Firmicutes.

### Concentrations of SCFA and BCFA in ileal contents

The total SCFA concentration was not significantly affected by diet. Of the individual straight-chain fatty acids and BCFAs tested, only propionate was influenced by any of the experimental factors, its concentration being significantly higher (*P* < 0.05) on day 45 of pregnancy than on day 75 (Table 6).

**Table 6:** Effect of diet and pregnancy stage on ileal SCFA and BCFA concentrations (mg/g) in Huanjiang mini-pigs.

Items	HN45	HN75	LN45	LN75	SEM	*P* values		
						Diet	Stage	Diet × Stage
Acetate	2.97	2.61	2.59	2.05	0.441	0.295	0.311	0.838
Propionate	0.31	0.16	0.24	0.17	0.134	0.429	0.016	0.339
Isobutyrate	0.06	0.06	0.06	0.05	0.076	0.757	0.991	0.740
Butyrate	0.39	0.33	0.23	0.17	0.184	0.055	0.482	0.993
Isovalerate	0.11	0.09	0.10	0.09	0.094	0.864	0.537	0.989
Valerate	0.05	0.03	0.02	0.02	0.064	0.062	0.453	0.241
A/P	11.67	15.44	11.79	12.47	1.091	0.600	0.416	0.569
BCFA	0.16	0.15	0.16	0.14	0.119	0.810	0.698	0.887
Straight-chain fatty acids	3.72	3.13	3.08	2.41	0.487	0.212	0.248	0.940
BCFA/Straight-chain fatty acids	0.04	0.06	0.05	0.06	0.068	0.961	0.218	0.675
Total SCFAs	3.88	3.29	3.24	2.56	0.493	0.225	0.257	0.936

A/P: acetate/propionate. BCFAs comprised isobutyrate and isovalerate; straight-chain fatty acids comprised acetate, propionate, butyrate, and valerate.

## Discussion

Owing to the potentially important roles of the intestinal microbiota in swine health and growth performance, its composition and metabolic activities in various physiological and nutritional contexts deserve close attention [28]. Changes in the composition of the intestinal (including bacterial profiles of the digesta and mucosa) and fecal microbiota have been demonstrated in pigs from birth to finishing phases [29–30] and in pregnant animals [31]. To the best of our knowledge, the present study represents the first analysis of changes in the ileal luminal bacterial profiles of Huanjiang mini-pigs.

In this study, on average, 39,895 effective reads were obtained for each sample, with high coverage (>99.08%). In general, alpha diversity indices were not influenced by diet or pregnancy stage. Coverage was marginally lower on day 45 of pregnancy compared to day 75. However, this difference was not statistically significant. The PCoA of overall diversity indicated that differences among individual pigs became greater as gestational age increased, as did indices of alpha diversity. The ileal microbial communities of pregnant Huanjiang mini-pigs were dominated by Firmicutes (69.99–82.22% of the total microbial content) and Proteobacteria (5.82–15.17%). This observation was consistent with the findings of Isaacson and Kim [32]. In the present study, *Clostridium* (11.00–21.75%), *Lactobacillus* (4.82–29.83%), and *Turicibacter* (12.47–19.16%) were the dominant bacterial genera in the ileal contents of Huanjiang mini-pigs, in accordance with previous surveys of the porcine ileal digesta- and mucosa-associated microbiota [33]. Collectively, these data support the assertion that Firmicutes constitutes the dominant phylum in the gut microbiota of mammals, including mice and humans [34].

*Lactobacillus* species are known for their potentially beneficial effects on gut function and health [35]. In our experiments, species of this genus were relatively more common in sows fed the HN diet (containing 11% soybean meal, a highly digestible plant protein source), which is consistent with a previous report that pigs fed normal levels of protein exhibit a greater abundance of *Lactobacillus* in the cecum compared with those given lower-protein feed [36]. Our previous *in vitro* studies indicated that soybean oligosaccharides (SBOS), major bioactive components of soybean meal, can be selectively fermented by commensal bacteria present in the colon, thus improving gut microbiota balance and modulating metabolism [37]. Dietary SBOS supplementation increases SCFAs, but decreases protein-derived catabolites in the intestinal luminal contents of weaned Huanjiang mini-piglets, which may have beneficial effects on the gut [27]. In addition, our results agree with a previously published report describing elevated numbers of lactobacilli in the ilea of pigs given a barley-based diet compared to those fed primarily on corn [28]. At the OTU level, the presence of OTU-45 (species: *Lactobacillus delbrueckii*) and OTU-906 (genus: *Lactobacillus*) decreased from day 45 to day 75 of pregnancy. *Lactobacillus* species have been associated with weight change in humans and animals [38]. However, the mechanism by which these microbes induce body weight loss or gain remains unclear.

Regarding other Firmicutes taxa, *Megasphaera* abundance was slightly lower in pigs fed the HN diet than in those given LN feed. Overall, our findings are similar to those of Pedersen *et al*. [39], who found bacteria of this phylum to be more abundant in the terminal ilea of obese pigs. Moreover, excess energy intake, obesity, and glucose intolerance are associated with increased presence of Firmicutes in humans [40,41].

In the present study, OTU-1, OTU-2, OTU-4, OTU-5, OTU-8, OTU-12, and OTU-1376 were among the 10 OTUs whose relative abundances distinguished, to a certain extent, species composition under different experimental conditions, i.e., diet group and pregnancy stage. All of these belonged to the phylum Firmicutes. In addition, considering it as a single indicator, this phylum was more affected by pregnancy stage than by diet.

Representatives of the phylum Proteobacteria were significantly less abundant than those of Firmicutes. Proteobacteria presence tended to be increased in the later stage of pregnancy (day 75). Koren *et al*. [15] showed that the relative abundance of Proteobacteria in fecal samples from pregnant women is higher in the third trimester of pregnancy than in the first. Moreover, a significant increase in the abundance of Proteobacteria has been associated with gastrointestinal inflammation in response to environmental and genetic factors [42], as observed in inflammation-associated dysbioses [43]. Shin *et al*. reported that members of Proteobacteria constitute a microbial signature of gut microbiota dysbiosis [44]. In the present study, this phylum was prominent and primarily represented by the genus *Sutterella* (0.20–1.48%). The abundance of OTU-10, a family (Enterobacteriaceae) within Proteobacteria, was also higher on day 75 than on day 45 of pregnancy. Several studies reported that active inflammatory bowel disease is associated with significantly elevated levels of Proteobacteria (members of Enterobacteriaceae in particular) [45]. Therefore, our results are compatible with suggestions that the structure and composition of bacterial communities in pregnant hosts are reminiscent of disease-associated dysbiosis.

Previous investigations indicated an association between raised Firmicutes/Bacteroidetes ratios and obesity [46]. *Clostridium* bacteria are also suspected to play a role in energy harvesting because they are found at higher levels in obese individuals than in people with low body weights [35]. Our prior study showed that the average back-fat thickness of pregnant Huanjiang mini-pigs in both HN and LN diet groups increases from day 45 (27.20 and 26.90 mm, respectively) to day 75 (36.60 and 28.10 mm, respectively) post-service [18]. The live body weights of sows in these treatment groups also increase over this period, from 73.82 and 67.52 kg to 86.14 and 75.28 kg, respectively (Kong et al., unpublished data). These data suggest that the sows became obese during pregnancy, especially those in the HN group. In the present work, the level of Firmicutes tended to be lower on day 75 than on day 45 of pregnancy. Bacteroidetes levels were stable between the two measured time points in the HN diet group, but were higher during the later stage of pregnancy in sows fed the LN diet. Therefore, the Firmicutes/Bacteroidetes ratio was lower on day 75 than on day 45 of pregnancy in both diet groups.

The presence of *Prevotella* species positively correlates with the proportion of carbohydrates in the diet. In a previously published study in which samples were clustered according to the prevalence of dietary components, representatives of this genus were found to be more abundant in a “carbohydrate” than in a “fat-protein” cluster [47]. In the current investigation, ileal levels of OTU-184 (genus: *Bacteroides*) tended to be higher on day 75 than on day 45 of pregnancy in Huanjiang mini-pigs. Bacteria of the genus *Prevotella* were more abundant in pigs fed the LN diet, whereas pregnancy stage did not significantly affect their numbers. This is consistent with observations of elevated *Prevotella* levels in goats fed a diet with reduced grain content [48]. In summary, the ratio of *Bacteroides* to *Prevotella* was less affected by the LN diet than by the HN diet. The abundance of Proteobacteria and Bacteroidetes in the ileal contents samples was higher on day 75 than on day 45 of pregnancy, suggesting an increase in these bacteria between the first and second trimesters.

Together, the results of the present study showed that bacteria of the phylum Firmicutes and genus *Lactobacillus* decreased in number, while those of the phylum Proteobacteria, family Enterobacteriaceae, and genus *Bacteroides* increased from day 45 to 75 of pregnancy. These changes in the gut microbiota are similar to those observed in inflammatory bowel disease, during which, the numbers of several species within Firmicutes are reduced, including those of *Lactobacillus*. Moreover, Enterobacteriaceae is among the Proteobacteria families whose levels appear to be consistently increased in this condition [49,50].

SCFAs are produced by the microbiota of the large intestine from both indigestible carbohydrates [51] and certain amino acids originating from partially digested dietary and endogenous proteins [52]. SCFAs regulate colonic physiology, metabolism, and gene expression [53]. They are also produced in the small intestine, but at concentrations lower than those in the colon, with the exception of acetate [5]. These molecules are produced via fermentation of indigestible polysaccharides by saccharolytic bacteria [54]. For example, species of *Ruminococcus*, *Oscillospira*, *Clostridium*, and *Pseudobutyrivibrio* metabolize fiber, while those of *Prevotella* metabolize hemicellulose, producing acetate and propionate [55]. In our study, the concentration of propionate in the ilea of sows was higher on day 45 than day 75 of pregnancy, which may be related to significant changes in *Bacteroides* abundance during this period. Notably, propionate is known to inhibit the synthesis of lipids from acetate [56].

In the large intestine, concentrations of BCFAs, bacterial metabolites produced exclusively from amino acids, are generally lower than those of SCFAs [57]. BCFAs such as isobutyrate and isovalerate are breakdown products of fermentation by proteolytic bacteria, including members of *Bacteroides* and *Clostridium* [58]. Various *Clostridium*, *Bacillus*, *Lactobacillus*, and *Streptococcus* species as well as many Proteobacteria species play major roles in the utilization of amino acids by their hosts [59].

## Conclusions

The ileal bacterial profiles of Huanjiang mini-pigs were dominated primarily by Firmicutes and Proteobacteria, and, in particular, representatives of the genera *Lactobacillus*, *Clostridium*, and *Turicibacter*. The effects of varying animal feed on the composition and diversity of the large intestinal microbiota also need to be elucidated, considering their potential importance. The HN diet was associated with a higher *Lactobacillus* abundance in pregnant Huanjiang mini-pigs. Since this diet differs from the LN diet in various characteristics (more digestible energy, higher relative protein content, and lower relative crude fiber content), it was not feasible in the current study to identify one particular dietary component associated with modifications in bacterial communities. In addition, the rice bran fiber and alfalfa used in both diets, but in inverse proportions, are characterized by different fiber type compositions [60,61]. Members of the phylum Firmicutes and genus *Lactobacillus* decreased, while those of the phylum Proteobacteria, family Enterobacteriaceae, and genus *Bacteroides* increased in number from day 45 to 75 of pregnancy. Notably, changes in bacterial community structure (e.g., increased number of Proteobacteria) as pregnancy progressed were similar to those observed in disease-associated dysbiosis (e.g., morbid obesity), indicating the need for further studies on a possible causal link between these parameters.

## Supporting information

S1 Table Effect of freeze-drying on the concentration of organic acids in biological samples (n = 9). (DOC)

S2 Table Raw reads and selected effective sequences in each group. (DOC)
