# Supplementary material for: Effects of dietary nutrient levels on microbial community composition and diversity in the ileal contents of pregnant Huanjiang mini-pigs
Source: PLoS One. 2017 Feb 14;12(2):e0172086. doi: 10.1371/journal.pone.0172086 (PMC5308767; doi:10.1371/journal.pone.0172086)
Supplement: S1 Table — (DOC) [file pone.0172086.s001.doc]

**S1 Table. Effect of freeze-drying on the concentration of organic acids in biological samples (n=9).**

| Items | Acetate | Propionate | Isobutyrate | Butyrate | Isovalerate | Valerate |
| --- | --- | --- | --- | --- | --- | --- |
| Freeze-dried samples | 3.71±0.19^b^ | 2.12±0.19 | 0.14±0.02 | 1.34±0.11 | 0.25±0.04 | 0.30±0.05 |
| Wet samples | 4.72±0.40^a^ | 2.34±0.24 | 0.14±0.02 | 1.34±0.11 | 0.22±0.03 | 0.27±0.04 |
