# Supplementary material for: Effects of dietary nutrient levels on microbial community composition and diversity in the ileal contents of pregnant Huanjiang mini-pigs
Source: PLoS One. 2017 Feb 14;12(2):e0172086. doi: 10.1371/journal.pone.0172086 (PMC5308767; doi:10.1371/journal.pone.0172086)
Supplement: S2 Table — (DOC) [file pone.0172086.s002.doc]

**S2 Table. Raw reads and selected effective sequences in each group.**

| Items | HN45 | HN75 | LN45 | LN75 | SEM | *P* values | | |
| --- | --- | --- | --- | --- | --- | --- | --- | --- |
|  |  |  |  |  |  | Diet | Stage | Diet × Stage |
| Raw reads | 43,195.4 | 42,229.5 | 41,653.25 | 41,356.5 | 504.02 | 0.264 | 0.555 | 0.754 |
| Effective sequences | 41,167.6 | 40,116.33 | 39,601 | 39,007.67 | 484.78 | 0.195 | 0.419 | 0.821 |

HN45 and HN75: data from samples obtained from Huanjiang mini-pigs fed a high-nutrient diet for 45 d and 75 d, respectively. LN45 and LN75: data from samples obtained from Huanjiang mini-pigs fed a low-nutrient-diet for 45 d and 75 d, respectively.
